# Binding of Dopamine to Alpha-Synuclein is Mediated by Specific Conformational States

**DOI:** 10.1007/s13361-013-0676-z

**Published:** 2013-07-02

**Authors:** Eva Illes-Toth, Caroline F. Dalton, David P. Smith

**Affiliations:** Biomedical Research Centre, Sheffield Hallam University, Sheffield, S1 1WB United Kingdom

**Keywords:** Ion mobility spectrometry, Protein folding, Amyloid, Dopamine, Parkinson’s disease

## Abstract

Parkinson’s disease is the second most common neurodegenerative disorder, in which both alpha-synuclein (α-syn) and dopamine (DA) have a critical role. α-Syn is known to be natively unstructured in equilibrium with subpopulations of more compact structures. It is these compact structures that are thought to be linked to amyloid formation. In the presence of DA, α-syn yields a diverse range of SDS-resistant, non-amyloid oligomers, however the precursor state conformation has not been established. Here, three DA molecules have been observed to bind per α-syn monomer by electrospray-ionization-ion mobility spectrometry-mass spectrometry (ESI-IMS-MS). Each of these DA molecules binds exclusively to the extended conformation of α-syn, and binding is not observed in the compact state of the protein. Measurements of collisional cross sectional areas show that the incremental uptake of DA pushes the protein towards a highly extended population, becoming fully populated upon the binding of three DA ligands. Tyrosine (Tyr) as a closely related structural analog, exhibited limited binding to the protein as compared with DA, with a maximum of two ligands being observed. Those Tyr ligands that do bind were observed as adducts to the extended conformation akin to DA. These findings suggest DA is able to modulate α-syn self-assembly by inducing the population of a highly extended state.

**Electronic supplementary material:**

The online version of this article (doi:10.1007/s13361-013-0676-z) contains supplementary material, which is available to authorized users.

**Figure:**
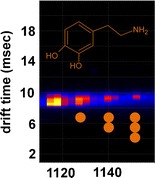
ᅟ

## Introduction

Alpha-synuclein (α-syn) is an intrinsically disordered protein that is closely associated with Parkinson’s disease (PD) and other neurodegenerative disease states [1]. The selective loss of dopaminergic neurons and the deposition of Lewy bodies (LBs) mainly comprised of fibrillar α-syn in the substantia nigra pars compacta are characteristic hallmarks of PD [1]. Oxidative stress has been shown to be one of the key mechanisms involved in neurotoxicity, giving a possible explanation as to why dopaminergic neurons are highly vulnerable to cell death: in the cytoplasm, dopamine (DA) can undergo oxidation because of its labile quinone ring in the presence of molecular oxygen producing reactive oxidative species (ROS) and dopamine quinones. ROS then can account for the damage in the cellular components, for example in the mitochondria through association of α-syn with oxidized lipids [2]. Since α-syn is thought to play an essential role in the modulation of synaptic vesicle recycling, DA storage and release at the nerve terminals [3], mutations in the protein in the familiar forms of the disease or changes in its expression or aberrant folding in the sporadic forms can consequently result in impaired neurotransmitter storage or release contributing to PD. In case of a breakdown in vesicle handling, an increased level of cytoplasmic DA may initiate damage by the production of free radicals and the formation of toxic intermediates with α-syn [4].

Recent research has focused on revealing the biochemical and molecular interactions of α-syn and DA with implications on the rate of secretion of α-syn-DA oligomers to the extracellular space, mitochondrial respiration and autophagy, respectively [5, 6]. In vitro, oligomers are devoid of a defined secondary structure, do not bind the amyloid specific dye thioflavin T, exhibit various non-ordered aggregate morphologies with alternate size and shape under electron microscopy and are sodium-dodecyl-sulphate resistant [7]. Some early evidence has indicated that oxidated ligation of DA stabilized α-syn yielding accumulation of DA- α-syn protofibrils [8]. These α-syn-DA oligomeric forms had an altered ability for self-assembly and propensity to disrupt biomembranes in a yeast model and may also be critical in sustaining neurodegeneration in PD [9, 10]. Structurally, α-syn is a natively disordered, 14,460 Da presynaptic protein that contains some ordered regions and can take on a variety of conformations depending on its immediate environment and the presence of binding partners [11]. It is known to co-populate both extended conformational states in equilibrium with more compact conformation states [12, 13]. Long range interactions between the C-terminal region and central regions of the protein have been reported by both nuclear magnetic resonance (NMR) and molecular dynamics (MD) simulations and as such the protein although unstructured has a conformation more compact than would be expected of a random coil [14, 15]. Alterations in solution conditions such as pH, the presence of metals or other binding ligands are known to increase the population of the compact conformation, correlating with increased aggregation rates as shown both by spectroscopy [16–18] and in the gas phase by IMS-MS [12, 13, 19]. Changes in solution conformation were detected in the gas phase as changes in collisional cross sectional area (Ω) consistent with a shift towards globular like assemblies and an increased intensity of lower charge state ions. The change in the charge state distribution occurs due to a decrease in available surface area able to take up H^+^ upon electrospray [20–22]. In a study undertaken by Bernstein et al. [12] it was shown that increasing injection energies (30, 50, 100 eV) resulted in increasing arrival time distributions of the compact conformation as the protein unfolded in the gas phase because of increasing energy. A shift to the more compact forms of α-syn as a result of acidification was also demonstrated in this study with an increased population of the compact state being observed [12]. Polyvalent metals (i.e., Cu^2+^, Fe^3+^, Co^3+^, and Mn^2+^) enhance the ability of α-syn to aggregate and can lead to increased rates of fibril formation in vitro [18, 23–25]. The resultant fibrils induced by the various cations have a particular morphology and degree of toxicity [23, 26]. To better understand the residue specific effects of metal-induced fibrillogenesis and structural transitions of monomeric α-syn in response to copper binding, a number of mapping studies have been undertaken employing circular dichroism (CD), calorimetric titrations, NMR, and electron paramagnetic resonance (EPR) spectroscopy [27–29], demonstrating the population of a compact species in the presence of this metal. This shift to a more compact conformation on metal binding has also been observed by ESI-MS, where again an increased population of the compact state was observed on co-ordination of copper [19]. These results indicate that changes in solution condition leading to conformational change [12, 30–33] can be subsequently observed in the gas phase [34] and suggest a role for the collapsed partially folded state as the precursor to amyloid formation akin to other amyloid systems [35, 36].

In order to investigate these conformational states in the context of DA binding, we harnessed the potential of ESI-IMS-MS as a platform to provide structural information in the form of Ωs [37] and ligand binding through mass shifts. The use of this technology in structural studies has been documented [38, 39]. Here we demonstrated through measurements of Ωs that the incremental uptake of the DA pushes α-syn towards a highly extended state, becoming fully populated upon the binding of three DA ligands. DA bound uniquely to the most extended conformations of α-syn indicating that the ligand binding site requires an extended conformation. The population of this DA bound extended state may represent the initial precursor state conformation to the soluble DA induced oligomers over the more amyloidogenic compact state. Tyr as a closely related structural analog to DA displayed a much reduced binding affinity to α-syn with a maximum of two ligands being bound by the protein and as far as we are aware has also not been reported to induce oligomerization of α-syn. Those Tyr ligands that do bind appear to bind to the same extended conformational state as the two DA ligands. Binding of the third DA ligand may therefore be key in switching pathways between amyloid-like aggregates and non-amyloid DA induced higher order oligomers, however further experimental data would be required to confirm this theory.

## Materials and Methods

### Materials

Equine cytochrome *c*, horse heart myoglobin, bovine ubiquitin, DA hydrochloride, glycine (Gly), α-Tyr, and ammonium acetate were purchased from Sigma-Aldrich (Gillingham, UK). Human, recombinant wild type α-syn was expressed in *E. coli* BL21 (DE3) and purified as described previously [40]. The concentration of pure monomeric α-syn was determined using the molar extinction constant 5960 M^−1^ cm^−1^ at A_280_ on a Jenway spectrophotometer.

### Sample Preparations

Protein samples for ESI-IMS-MS experiments were prepared by dissolving α-syn to a 40 μM final concentration and by diluting DA or Tyr to a 6.25 mM final concentration in aqueous solution of 50 mM ammonium acetate pH 6.8. The following protein to ligand ratios of the working strength solutions were analyzed: 1:125, 1:30, and 1:8 (α-syn:DA), all prepared and mixed immediately prior to analysis. The experiments were conducted either in the presence or absence of 6-fold excess of Gly to avoid nonspecific interactions.

### ESI-IMS-MS Analysis

All spectra were acquired using a Synapt G2 HDMS instrument (Waters, Manchester, UK) by use of gold coated home-made borosilicate nano-capillaries in positive mode. Optimized instrumental settings for data acquisition were: capillary voltage of 1.70–1.90 kV, cone voltage of 50 V, source temperature of 60 °C, trap collision energy of 4.0 V, transfer collision energy of 10 V, trap bias 45, backing pressure of 3.1 mbar. IMS separations were performed at T-wave velocities of Trap:311, IMS:800 and Transfer:200 m/s and T-wave amplitudes of 4–15 V using 3.6 mbar pressure of nitrogen gas maintained by a 90 mL/min gas flow. Calibration curve for collisional cross sections was obtained based on of multiple charge states of equine cytochrome *c*, horse heart myoglobin and bovine ubiquitin (Sigma Aldrich) as described previously [41]. For attaining denatured spectra, all calibrants and mass standards were prepared before injection at 10–15 μM and α-syn at 10 and 40 μM and dissolved in 10 % formic acid, 50 % acetonitrile and 40 % ultrapure water (vol/vol/vol), whilst instrumental settings were maintained as mentioned before. Mass calibration was carried out by an infusion of CsI cluster ions, and arrival time distributions were determined by using the Mass Lynx v4.1 software (Waters).

## Results and Discussion

**Figure 1:**
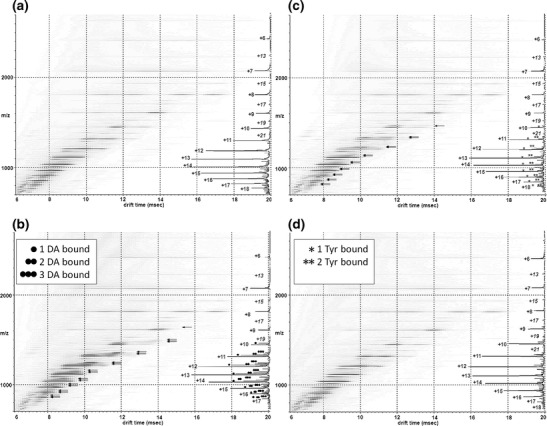
Driftscope plots obtained from 50 mM aqueous ammonium acetate solution of 40 μM α-syn, at pH 6.8 with or without further addition of either DA or Tyr at 6.25 mM. The x axis represents drift time (ms), on the y axis *m/z* is shown; square root display. Corresponding mass spectrum for each acquisition is overlaid on the right of each Driftscope plot indicating charge states and the presence or absence of the appropriate ligands (**a**) wild-type α-syn, (**b**) α-syn in the presence of DA, (**c**) α-syn in the presence of Tyr, (**d**) α-syn in the presence of DA and Gly

**Figure 2:**
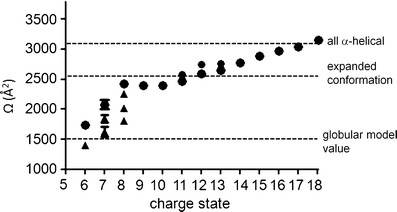
Conformational series of α-syn. Filled, black circles: extended conformational features, black triangles: compact conformational forms. The dotted lines represent theoretical Ωs calculated for structures having an all α-helical or globular conformation along with the Ω for the extended conformation as reported by Bernstein et al. [12]. Neither of these calculated or estimated values are true representations of the protein’s molecular organization; they have been adopted and superimposed on our experimental data to aid comparison. Experimentally measured values represent three independent measurements taken on alternate days using independent protein preparations

ESI-IMS-MS analysis was performed utilizing a Waters Synapt G2 in positive ion mode. Typical tuning parameters were initially varied extensively in order to investigate and minimize any artifacts within the data. A range of capillary (1.5 to 3.0 kV) and cone voltages (40–100 V) along with changes in backing pressures between 1.0 and 7.0 mbar and trap, transfer (4–20), bias (45–100) and voltages along with wave guide parameters were optimized. Final experimental parameters are listed in the Section 2. Supplementary Figure 1 shows Ωs of known globular proteins with similar mass to α-syn acquired under our experimental conditions. The Ωs of these known proteins are very close to those previously reported [41] and well within theoretically calculated [42, 43] values. Our ESI-IMS-MS analysis of wild-type α-syn revealed a primarily extended population (charge state ions +18 to +8) and a subpopulation of a more compact conformational series (charge state ions +8 to +6) with multiple overlapping features in both series and a less observable dimeric species under native conditions (Figure 1a). Figure 2 outlines the resolvable Ω of the individual charge state ions observed under native conditions. Within the extended series three charge state ions +11, +12, and +13 show at least two major resolvable conformations. Since these charge state represented the most intense peaks within the spectra it is possible that the other charge state ions also have multiple features but we were unable to detect them due to either low intensity or resolution. Alternatively, the larger Ω of the states could be an artefact induced by Coulombic repulsion due to the high charge on the molecule. Another alternative explanation is that there are two major distinguishable conformational families present within the extended conformations, the first encompassing charge state ions +13 to +8 and the second encompassing charge state ions +18 to +11, these two major states would therefore have the +13 to +11 charge states in common. This theory is consistent with the charge state distribution analysis of Frimpong et al. [13] detailed below.

It is known that compact states in solution when subjected to ESI acquire fewer charges and have a narrow charge state distribution, whereas unfolded states acquire a large number of charges and have a wide charge state distribution [20, 21]. The standard MS data presented in Figure 1a and Supplementary Figure 2 clearly showed multiple charge state distributions centered at the +13 and +7 charge state ions. Both the +7 and +8 charge state ions were more intense than would be expected for a completely unfolded protein. This charge state distribution is consistent with known data for globular proteins and, given the mass of the protein (14.6 kDa), we would predict a narrow distribution centered on the +7 charge state ion [20–22] as observed here. In order to further confirm that the compact conformation reflected that present in solution and did not arise solely because of transfer to the gas phase, an α-syn mass spectrum was acquired under fully denaturing conditions (50 % acetonitrile, 40 % H_2_O, and 10 % formic acid) Supplementary Figure 3. If the lower charge state ions of the protein underwent collapse during acquisition, it would be expected that this is observed regardless of the solution conditions from which the protein was acquired. Charge state ions between +19 and +8 were observed for fully denatured α-syn with no evidence of a significant compact conformation at the lower charge state ions akin to that seen under native conditions. In addition, the intensity of the charge state ions +9 to +8 were markedly lower than that observed under native conditions, indicating a single extended conformation. In comparison with other published data, Frimpong et al. ascertained four states of the protein in the pH range of 4–8: an extended form (U) at high charge states (+21 to +13 charge state ions), I_1_ representing helix-rich intermediates (+16 to +10 charge state ions), I_2_ entailing β-rich intermediate state (+13 to +7 charge state ions), and a highly compact forms spanning low charge states (+9 to +6 charge state ions) [13]. In negative ion mode, a study by the Bowers group reported two main conformational families for wild type A53T and A30P α-syn, one dominating the lower charge states (−16 to −6 charge state ions) centered at −11 with multiple features and one prevalent at high charge states (−8 to −6 charge state ions) [34]. The compact state of α-syn was modeled by a globular structure ~1500 Å^2^, whereas the extended state had a reported Ω of 2530 Å^2^ [12] with a spread between ~1800 Å^2^ and 3000 Å^2^ over all charge states [34]. The extended state was shown to be more compact than a fully random coil conformation as predicted by small angle X-ray scattering (SAXS) and NMR [16]. These model and reported Ω values have been taken from these publications [12, 43] and plotted onto Figure 2 in order to compare the experimental data acquired here against both model and previously reported values. Ωs of each of the charge states and conformational families observed here are in agreement with these previously reported values [12, 34] and the Ω of the compact state of α-syn observed is comparable both theoretically (Figure 2) and experimentally (Supplementary Figure 1 and Table 1) to globular proteins of a similar mass [13, 34, 41, 42, 44].

Numerous in vitro studies have shown that DA and some of its metabolites act as modulators of α-syn oligomerization and inhibit its fibrillization [7, 8, 45, 46]. ESI-MS in conjunction with other biophysical methods has demonstrated that one intact, unoxidized α-syn is capable of binding 3 DA molecules that in turn led to oligomerization of a range of higher order protofibril species [47]. Here, the effect of DA binding on the population of conformational states was examined in conjunction with changes in the Ωs. α-Syn-DA complexes observed by ESI-IMS-MS are shown in Figure 1 and the ESI-MS spectra of apo α-syn, α-syn-DA, α-syn-Tyr, and α-syn-DA-Gly is included as Supplementary Figure 2. DA ligands are highlighted with black dots on the mass spectrum in Figure 1b and are only observed on the +17 to +10 charge states. If the extended state was the sole conformation present or DA binding was nonspecific, then it might be expected that DA adducts would be present on all charge state ions (+6 to +17) in the gas phase. However, binding of 3 DA could be only observed to the +11 to +17 charge state ions in the same envelope that contains the most extended forms of the protein. The +10 charge state ion bound only a single DA ligand. The lower charge state ions +6 to +9 did not show any DA binding and these ions in the absence of DA still retained the structural features of the most compact conformation. It is possible that ligand binding stabilizes the extended state preventing structural collapse; however, the conformational states that the ligand binds appear to be present in the absence of the ligand. This data can, therefore, be interpreted as DA bound only to the most extended conformations in solution.

**Figure 3:**
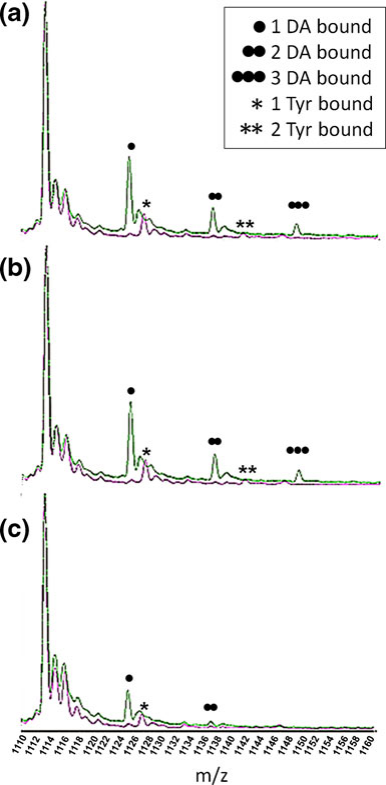
Binding of DA and binding of Tyr to α-syn at the +13 charge state ion. Spectra were acquired at protein to DA ratios of (**a**) 1:125, (**b**) 1:30, and (**c**) 1:8. Incrementally bound α-syn DA adducts (1–3) are marked with black circles, the *m/z* 1113.3 is the apo form of α-syn [M + 13H]^+13^. Stars illustrate the Tyr-α-syn adducts. X axis represents the *m/z* values and the y axis represents relative abundance in % of the base peak

Tyr serves as an early precursor in the synthesis of DA [48] and differs only by a hydroxyl group and a carboxyl group in its chemical composition. Figure 1c details the resultant α-syn-Tyr spectra obtained from a 1:125 ratio of α-syn:Tyr. Tyr adducts are marked with single or double stars according to the number of ligands bound. A maximum of two molecules of Tyr were involved in complex formation with α-syn indicated by one or two stars above the respective peaks; these ions, however, had a markedly lower intensity compared with apo- and DA-bound forms of α-syn (Figure 3). Again, ligand binding was only observed to the +17 to +11 charge state ions as for DA, and no binding was observed to the more compact conformational populations of α-syn. Inclusion of Gly is useful when preventing nonspecific or low affinity binding and here it completely negated the ability of DA to bind to α-syn as shown on Figure 1d, suggesting the interactions were low affinity and electrostatic in nature. Together, these results demonstrated that the extended conformations populating the +17 to +11 charge state ions of α-syn will readily bind up to 1–3 DA molecules, have a lower affinity for Tyr, and that this binding could be overcome by the presence of Gly.

**Figure 4:**
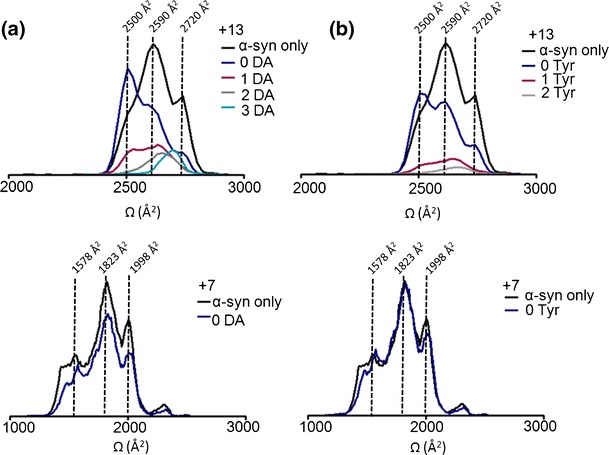
Ωs of incremental adducts of (**a**) DA and (**b**) Tyr bound to α-syn at the +13 and +7 charge state ions compared, in addition α-syn only acquired in the absence of ligand is shown The +13 and +7 charge state ions are highlighted as being representative of the extended and compact states, respectively. Alternate populations are indicated by vertical dotted lines with corresponding Ω areas. Black line: α-syn only, navy blue: apo-form of the protein remaining ligand-free in the presence of DA, purple: 1 DA bound, grey: 2 DA bound and light blue: 3 DA bound. No ligand binding was observed at the +7 charge state ion as highlighted with both a black line (α-syn only) and a navy blue line (remained ligand free in the presence of DA). (**b**) Tyr adducts of +13 and +7 charge state ions or unbound populations at the 1:125 ratio are shown. The black line indicates α-syn only, navy line shows the protein remained ligand-free, 1 Try bound protein is indicated by a purple line, the 2 Tyr bound form is denoted by a grey line. The +7 charge state ion remained ligand-free (navy blue line) at the 1:125 molar ratio overlapping the Ω of α-syn only (black line)

To further elucidate the conformational transitions taking place upon DA coordination, we concentrated on selected populations of the extended and compact conformational families of α-syn (Figure 4). We assigned the +13 charge state ion as being representative of the extended conformational family. The arrival time distribution for the +13 charge state ion acquired in the absence of any DA is shown in Figure 3a: black line and indicates two clearly resolvable peaks at ~2590 Å^2^ and ~2720 Å^2^ along with another less pronounced feature at ~2500 Å^2^. In the presence of DA, the most extended forms of the protein bound to the ligand with a shift toward the more extended states. The stepwise binding of 1, 2, or 3 DA ligands initiated a shift in the conformation towards the more extended conformations (Figure 4a) as evidenced by an increasing drift time and increase in the Ωs. Upon binding of 3 DA ligands, the ~2500 Å^2^ and ~2590 Å^2^ states were absent and the highly extended state, ~2720 Å^2^ was uniquely observed. The remaining apo-form of the protein acquired in the presence of DA predominantly showed the populations of the ~2500 Å^2^ conformations and significant depletion of the ~2590 Å^2^ and ~2720 Å^2^ conformations, presumably as these conformations were now ligand bound. The +7 charge state ion was taken as being representative of the compact states of α-syn and co-populated compact conformations along with a minor population of the extended state. DA binding was not observed to any of the compact conformations, nor a shift in Ω was observed. One or two Tyr ligands were also observed to bind to the extended states of α-syn charge states +17 to +11. Akin to DA, the binding was to the most extended forms of the protein. However, as Figure 3 demonstrates, this binding was much weaker compared with DA at the same protein to ligand ratios. Therefore, Tyr was able to bind to α-syn in a similar manner to DA but had a much reduced affinity. These results, therefore, demonstrated that DA binds to α-syn in a conformational dependent manner, binding exclusively to the most extended states of the protein.

**Figure 5:**
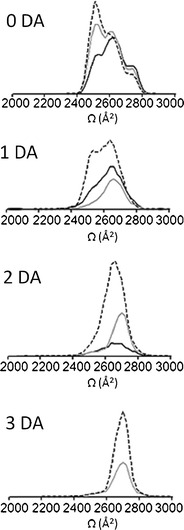
Ωs of α-syn co-ordinating DA or remaining ligand free at +13 charge state. Ωs are shown at three different protein to ligand molar ratios; 1:8 is marked with a black line, 1:30 is marked with a grey line and 1:125 is illustrated with a dashed line. The protein remained ligand-free in the presence of the ligand (0 DA) is depicted at the top then incremental adducts of α-syn-DA are placed sequentially below as follows: 1 DA-, 2 DA- and 3 DA-bound to α-syn at three alternate protein to DA molar ratios. Data was normalized to the most intense peak of the spectra

To assess that adduct formation and subsequent conformational rearrangement following DA ligation occurs through repeated measurements and at reduced dilutions yet still higher than physiological DA levels [49], we conducted replicate experiments at 1:125, 1:30, and 1:8 ratios (α-syn:DA). Up to 3 DA molecules were coordinated in each case, however, the intensity of the adduct peaks became lower as the ligand concentration decreased (Figures 3 and 5). Ratios of 1:125 and 1:30 were required to observe binding of 3 DA ligands to α-syn. The majority of the protein was present in the most extended states (~2720 Å^2^) upon binding of 3 DA molecules and at high ratios of α-syn:DA the ~2500 Å^2^ state was effectively absent. Replicate experiments have shown excellent agreement in calculated Ωs of the three independent measurements taken from different protein preparations. The alternate α-syn:DA ratios did not dramatically affect the absolute Ωs recorded of the extended state conformations, with 2 and 3 DA bound at low ratios inducing the same Ω as 2 and 3 DA bound at high ratios. These results would indicate that it is the specific binding of the ligand that causes the conformational change rather than a change in the solution environment due to the presence of the DA.

## Conclusions

Crucial insights have been gained into how DA might be involved in aggregation and the fibrillization of α-syn from in vitro studies [7–9, 46] and evidence now emerges that polymers of α-syn-DA or other reactive intermediates are linked to neuronal cell death in PD [6]. Although the co-ordination of DA to α-syn has been observed before [47], here we have shown that coordination of three DA molecules to wild-type α-syn induces a highly extended conformation specifically binding to the extended states and causes an increase in Ω. No binding of DA was observed under our conditions to the compact states. This concept of expansion is not dissimilar from that which Rekas et al. have reported. In their study, α-syn was incubated for 5 to 7 d in the presence of DA, after which the protein was observed to be oxidized. The resulting Met-oxidized α-syn monomer and α-syn-DA trimer had elongated shapes versus untreated monomers based on SAXS CD results, which could be brought about by the association of the DA bound extended states observed here prior to incubation. However, there are marked differences between the experimental conditions used in the study of Rekas et al. and our own buffer conditions and protein concentration [50]. Met-oxidation has been observed to be required for DA induced oligomerization [51, 52]. Utilizing short time frames (<5 min) we did not observe extensive Met-oxidation of α-syn. Oxidation was only seen at the highest charge states and were predominantly lower intensity adducts beside the more pronounced, unoxidized higher intensity DA-bound peaks and ligand-free peaks. However, minor oxidation was present without any prior addition of ligand and, hence, did not result from DA exposure. Longer incubations (>12 h) of α-syn in the presence of DA did result in oxidation of the monomeric protein (Supplementary Figure 4) akin to other studies [53]. Regardless of this observation, during the time frame of our experiments, the unoxidized α-syn-DA complex was considerably more dominant, demonstrating oxidation is not required for initial binding and further oxidation is a result of long-term exposure to DA.

Alternate ligands have been reported to bind to α-syn by a range of mass spectrometry methods each affecting the conformational state of the monomer in its own way. Electrospray ionization-electron capture dissociation-mass spectrometry/mass spectrometry (ESI-ECD-MS/MS) has been used in probing non-covalent protein-ligand binding interactions in relation to α-syn. Spermine is a polyamine compound widely found in many tissues and has a role in increasing the aggregation rate of α-syn [30]. The spermine binding site has been localized to the C-terminal 106–138 amino acids residues of the wild-type α-syn, as demonstrated by Xie et al. [54] using ESI-ECD-MS/MS, consistently with previous NMR data [31]. Changes in the charge state distribution were, however, not observed in this study. The DA binding site of α-syn is also located on the highly negatively charged C-terminal region of the protein, at ^125^YEMPS^129^; this sequence has been implicated in DA interactions and in modulation of DA induced aggregation [45]. This region, however, has been reported to be necessary only for oxidation but not complete binding [52]. Interestingly and similarly to the DA binding results here, data presented in the Supplementary Information of the paper by Xie et al*.* showed the 1:2 protein–ligand complex binding only to the high charge state ions (+18 to +11 charge state ions) [54]. ESI-IMS-MS has also been used to gain conformational information for the familial point mutation of α-syn A53T upon spermine binding. Evidence from NMR data suggests that although spermine binds specifically to the C-terminal of α-syn, it induces changes in the N-terminal in the proximity of Gly and Tyr residues in the region of amino acids 22–93 and leads to the adoption of a β-sheet conformation typical of fibrillar α-syn [31]. Spectra acquired in negative ion mode by Grabenauer et al. [34] reported a significantly greater percentage of the population in the compact form in the spermine:protein complex as compared to the unbound protein. Either the presence of this point mutation is involved in the binding of spermine through long-range interactions or its inclusion facilitates aggregation through preferential population of the compact state.

Here, the shift in the population towards a more extended state on DA binding may well be the point at which the aggregation pathway is switched from the amyloid-forming pathway linked to the collapsed state [16, 19, 55] to the pathway resulting in soluble DA induced oligomers. Both DA and Tyr bind uniquely to the extended conformational state of α-syn, yet Tyr is not known to induce aggregation. The binding of the third DA ligand to the most extended state may well be the critical step in switching the aggregation pathways as only two Tyr ligands are observed to bind α-syn. However, further mutational experiments that limit the DA binding will be required to confirm this theory. From the data presented here we can conclude that DA first binds to extended conformations of the protein, followed by oxidation of Met residues [51, 52] resulting in oligomer formation. Previous SAXS data has demonstrated that DA induced α-syn oligomers on extended incubation had a highly extended conformation [50], and this structure may well result from the stabilization of a highly extended monomeric state.

We therefore propose that DA is a potent modulator of α-syn assembly and exerts its effect at the very early stages of aggregation by influencing the conformational dynamics of the monomeric protein.

## Electronic supplementary material

Below is the link to the electronic supplementary material.

ESM 1 (DOCX 248 kb)

## References

[1] Jellinger KA (2011). Interaction between alpha-synuclein and other proteins in neurodegenerative disorders. Sci. World J..

[2] Ruiperez V, Darios F, Davletov B (2010). Alpha-synuclein, lipids, and Parkinson’s disease. Prog. Lipid Res..

[3] Chadchankar H, Ihalainen J, Tanila H, Yavich L (2011). Decreased reuptake of dopamine in the dorsal striatum in the absence of alpha-synuclein. Brain Res..

[4] Lundblad M, Decressac M, Mattsson B, Bjorklund A (2012). Impaired neurotransmission caused by overexpression of alpha-synuclein in nigral dopamine neurons. Proc. Natl. Acad. Sci. U. S. A..

[5] Lee HJ, Baek SM, Ho DH, Suk JE, Cho ED, Lee SJ (2011). Dopamine promotes formation and secretion of non-fibrillar alpha-synuclein oligomers. Exp. Mol. Med..

[6] Outeiro TF, Klucken J, Bercury K, Tetzlaff J, Putcha P, Oliveira LM, Quintas A, McLean PJ, Hyman BT (2009). Dopamine-induced conformational changes in alpha-synuclein. PLoS One.

[7] Cappai R, Leck SL, Tew DJ, Williamson NA, Smith DP, Galatis D, Sharples RA, Curtain CC, Ali FE, Cherny RA, Culvenor JG, Bottomley SP, Masters CL, Barnham KJ, Hill AF (2005). Dopamine promotes alpha-synuclein aggregation into SDS-resistant soluble oligomers via a distinct folding pathway. FASEB J..

[8] Conway KA, Rochet JC, Bieganski RM, Lansbury PT (2001). Kinetic stabilization of the alpha-synuclein protofibril by a dopamine-alpha-synuclein adduct. Science.

[9] Li HT, Lin DH, Luo XY, Zhang F, Ji LN, Du HN, Song GQ, Hu J, Zhou JW, Hu HY (2005). Inhibition of alpha-synuclein fibrillization by dopamine analogs via reaction with the amino groups of alpha-synuclein. Implication for dopaminergic neurodegeneration. FEBS J..

[10] Rochet J, Outeiro TF, Conway KA, Ding TT, Volles MJ, Lashuel HA, Bieganski RM, Lindquist SL, Lansbury PT (2004). Interactions among alpha-synuclein, dopamine, and biomembranes: Some clues for understanding neurodegeneration in Parkinson’s disease. J. Mol. Neurosci..

[11] Uversky VN, Eliezer D (2009). Biophysics of Parkinson’s disease: Structure and aggregation of alpha-synuclein. Curr. Protein Pept. Sci..

[12] Bernstein SL, Liu D, Wyttenbach T, Bowers MT, Lee JC, Gray HB, Winkler JR (2004). Alpha-synuclein: Stable compact and extended monomeric structures and pH dependence of dimer formation. J. Am. Soc. Mass Spectrom..

[13] Frimpong AK, Abzalimov RR, Uversky VN, Kaltashov IA (2010). Characterization of intrinsically disordered proteins with electrospray ionization mass spectrometry: Conformational heterogeneity of alpha-synuclein. Protein.

[14] Breydo L, Wu JW, Uversky VN (2012). Alpha-synuclein misfolding and Parkinson’s disease. Biochim. Biophys. Acta.

[15] Dedmon MM, Lindorff-Larsen K, Christodoulou J, Vendruscolo M, Dobson CM (2005). Mapping long-range interactions in alpha-synuclein using spin-label NMR and ensemble molecular dynamics simulations. J. Am. Chem. Soc..

[16] Uversky VN, Li J, Fink AL (2001). Evidence for a partially folded intermediate in alpha-synuclein fibril formation. J. Biol. Chem..

[17] Uversky VN, Li J, Bower K, Fink AL (2002). Synergistic effects of pesticides and metals on the fibrillation of alpha-synuclein: Implications for Parkinson’s disease. NeuroToxicology.

[18] Manning-Bog AB, McCormack AL, Li J, Uversky VN, Fink AL, Di Monte DA (2002). The herbicide paraquat causes up-regulation and aggregation of alpha-synuclein in mice: Paraquat and alpha-synuclein. J. Biol. Chem..

[19] Natalello A, Benetti F, Doglia SM, Legname G, Grandori R (2011). Compact conformations of alpha-synuclein induced by alcohols and copper. Proteins.

[20] Fenn JB, Mann M, Meng CK, Wong SF, Whitehouse CM (1989). Electrospray ionization for mass spectrometry of large biomolecules. Science.

[21] Kaltashov IA, Mohimen A (2005). Estimates of protein surface areas in solution by electrospray ionization mass spectrometry. Anal. Chem..

[22] de la Mora JF (2000). Electrospray ionization of large multiply charged species proceeds via Dole’s charged residue mechanism. Anal. Chim. Acta.

[23] Brown DR (2010). Oligomeric alpha-synuclein and its role in neuronal death. IUBMB Life.

[24] Uversky VN, Li J, Fink AL (2001). Metal-triggered structural transformations, aggregation, and fibrillation of human alpha-synuclein. A possible molecular NK between Parkinson’s disease and heavy metal exposure. J. Biol. Chem..

[25] Brown DR (2007). Interactions between metals and alpha-synuclein—function or artefact?. FEBS J..

[26] Bharathi Indi SS, Rao KS (2007). Copper- and iron-induced differential fibril formation in alpha-synuclein: TEM study. Neurosci. Lett..

[27] Binolfi A, Lamberto GR, Duran R, Quintanar L, Bertoncini CW, Souza JM, Cervenansky C, Zweckstetter M, Griesinger C, Fernandez CO (2008). Site-specific interactions of Cu(II) with alpha- and beta-synuclein: Bridging the molecular gap between metal binding and aggregation. J. Am. Chem. Soc..

[28] Binolfi A, Rodriguez EE, Valensin D, D’Amelio N, Ippoliti E, Obal G, Duran R, Magistrato A, Pritsch O, Zweckstetter M, Valensin G, Carloni P, Quintanar L, Griesinger C, Fernandez CO (2010). Bioinorganic chemistry of Parkinson’s disease: Structural determinants for the copper-mediated amyloid formation of alpha-synuclein. Inorg. Chem..

[29] Drew SC, Leong SL, Pham CL, Tew DJ, Masters CL, Miles LA, Cappai R, Barnham KJ (2008). Cu2+ binding modes of recombinant alpha-synuclein-insights from EPR spectroscopy. J. Am. Chem. Soc..

[30] Antony T, Hoyer W, Cherny D, Heim G, Jovin TM, Subramaniam V (2003). Cellular polyamines promote the aggregation of alpha-synuclein. J. Biol. Chem..

[31] Fernandez CO, Hoyer W, Zweckstetter M, Jares-Erijman EA, Subramaniam V, Griesinger C, Jovin TM (2004). NMR of alpha-synuclein-polyamine complexes elucidates the mechanism and kinetics of induced aggregation. EMBO J..

[32] Binolfi A, Rasia RM, Bertoncini CW, Ceolin M, Zweckstetter M, Griesinger C, Jovin TM, Fernandez CO (2006). Interaction of alpha-synuclein with divalent metal ions reveals key differences: A link between structure, binding specificity and fibrillation enhancement. J. Am. Chem. Soc..

[33] Lee JC, Gray HB, Winkler JR (2008). Copper(II) binding to alpha-synuclein, the Parkinson’s protein. J. Am. Chem. Soc..

[34] Grabenauer M, Bernstein SL, Lee JC, Wyttenbach T, Dupuis NF, Gray HB, Winkler JR, Bowers MT (2008). Spermine binding to Parkinson’s protein alpha-synuclein and its disease-related A30P and A53T mutants. J. Phys. Chem. B.

[35] Chiti F, Dobson CM (2009). Amyloid formation by globular proteins under native conditions. Nat. Chem. Biol..

[36] Illes-Toth E, Smith DP (2013). Conformations and assembly of amyloid oligomers by electrospray ionization-ion mobility spectrometry-mass spectrometry. Curr. Anal. Chem..

[37] Zhong Y, Hyung SJ, Ruotolo BT (2011). Characterizing the resolution and accuracy of a second-generation traveling-wave ion mobility separator for biomolecular ions. Analyst.

[38] Woods, L.A., Radford, S.E., Ashcroft, A.E.: Advances in ion mobility spectrometry-mass spectrometry reveal key insights into amyloid assembly. *Biochim. Biophys. Acta*. **1834**, 1257–1268 (2013) 10.1016/j.bbapap.2012.10.002 PMC3787735 23063533

[39] Jurneczko E, Barran PE (2011). How useful is ion mobility mass spectrometry for structural biology? The relationship between protein crystal structures and their collision cross sections in the gas phase. Analyst.

[40] Smith DP, Tew DJ, Hill AF, Bottomley SP, Masters CL, Barnham KJ, Cappai R (2008). Formation of a high affinity lipid-binding intermediate during the early aggregation phase of alpha-synuclein. Biochemistry.

[41] Smith, D.P., Knapman, T.W., Campuzano, I., Malham, R.W., Berryman, J.T., Radford, S.E., Ashcroft, A.E.: Deciphering drift time measurements from travelling wave ion mobility spectrometry-mass spectrometry studies. *Eur. J. Mass Spectrom*. (Chichester, Eng) **15**, 113–130 (2009) 10.1255/ejms.947 19423898

[42] Shvartsburg AA, Jarrold MF (1996). An exact hard-spheres scattering model for the mobilities of polyatomic ions. Chem. Phys. Lett..

[43] von Helden G, Gotts NG, Bowers MT (1993). Experimental evidence for the formation of Fullerenes buy collisional heating of carbon rings in the gas phase. Nature.

[44] Cornell WD, Cieplak P, Bayly CI, Gould IR, Merz KM, Ferguson DM, Spellmeyer DC, Fox T, Caldwell JW, Kollman PA (1995). A second generation force field for the simulation of proteins and nucleic acids. J. Am. Chem. Soc..

[45] Norris EH, Giasson BI, Hodara R, Xu S, Trojanowski JQ, Ischiropoulos H, Lee VM (2005). Reversible inhibition of alpha-synuclein fibrillization by dopaminochrome-mediated conformational alterations. J. Biol. Chem..

[46] Jinsmaa Y, Florang VR, Rees JN, Mexas LM, Eckert LL, Allen EM, Anderson DG, Doorn JA (2011). Dopamine-derived biological reactive intermediates and protein modifications: Implications for Parkinson’s disease. Chem. Biol. Interact..

[47] Shimotakahara S, Shiroyama Y, Fujimoto T, Akai M, Onoue T, Seki H, Kado S, Machinami T, Shibusawa Y, Uéda K, Tashiro M (2012). Demonstration of three dopamine molecules bound to *α*-synuclein: Implication of oligomerization at the initial stage. J. Biophys. Chem..

[48] Felger JC, Li L, Marvar PJ, Woolwine BJ, Harrison DG, Raison CL, Miller AH (2012). Tyrosine metabolism during interferon-alpha administration: Association with fatigue and CSF dopamine concentrations. Brain Behav. Immun..

[49] Saha B, Mondal AC, Majumder J, Basu S, Dasgupta PS (2001). Physiological concentrations of dopamine inhibit the proliferation and cytotoxicity of human CD4+ and CD8+ T cells in vitro: A receptor-mediated mechanism. Neuroimmunomodulation.

[50] Rekas A, Knott RB, Sokolova A, Barnham KJ, Perez KA, Masters CL, Drew SC, Cappai R, Curtain CC, Pham CL (2010). The structure of dopamine induced alpha-synuclein oligomers. Eur. Biophys. J..

[51] Leong SL, Cappai R, Barnham KJ, Pham CL (2009). Modulation of alpha-synuclein aggregation by dopamine: A review. Neurochem. Res..

[52] Leong SL, Pham CL, Galatis D, Fodero-Tavoletti MT, Perez K, Hill AF, Masters CL, Ali FE, Barnham KJ, Cappai R (2009). Formation of dopamine-mediated alpha-synuclein-soluble oligomers requires methionine oxidation. Free Radic. Biol. Med..

[53] Chan T, Chow AM, Cheng XR, Tang DWF, Brown IR, Kerman K (2012). Oxidative stress effect of dopamine on α-synuclein: Electroanalysis of solvent interactions. ACS Chem. Neurosci..

[54] Xie Y, Zhang J, Yin S, Loo JA (2006). Top-down ESI-ECD-FT-ICR mass spectrometry localizes noncovalent protein-ligand binding sites. J. Am. Chem. Soc..

[55] Uversky VN, Lee HJ, Li J, Fink AL, Lee SJ (2001). Stabilization of partially folded conformation during alpha-synuclein oligomerization in both purified and cytosolic preparations. J. Biol. Chem..

