# Supplementary material for: Binding of Dopamine to Alpha-Synuclein is Mediated by Specific Conformational States
Source: J Am Soc Mass Spectrom. 2013 Jul 2;24(9):1346–54. doi: 10.1007/s13361-013-0676-z (PMC3738842; doi:10.1007/s13361-013-0676-z)
Supplement: Supplementary file 1 — (DOCX 248 kb) [file 13361_2013_676_MOESM1_ESM.docx]

Binding of dopamine to alpha-synuclein is mediated by specific conformational states.

Eva Illes-Toth, Caroline F. Dalton and David P. Smith*.

Biomedical Research Centre, Sheffield Hallam University, Sheffield, S1 1WB, UK

Supplementary Information


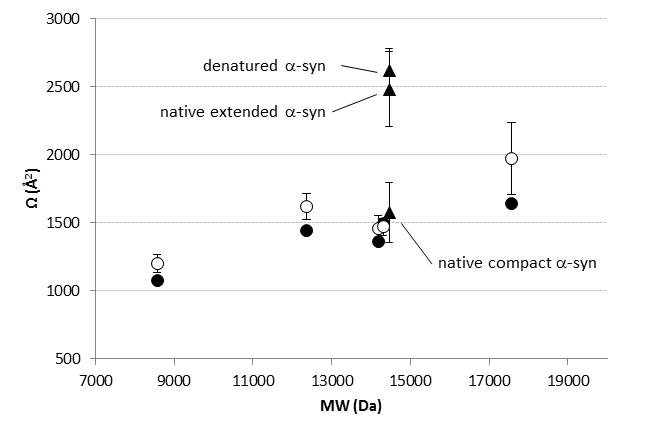


**Supplementary Figure 1.** Experimental Ω for bovine ubiquitin, equine cytochrome c, α-lactalbumin, hen egg lysozyme and horse heart myoglobin, native α-synuclein and denatured α-synuclein. Ωs are plotted as mean values across all charge states, error bars represent the spread in the recorded Ω. White circles represent the experimental values acquired here, black circles represent previously published Ω values [1], black triangles represent the native extended and compact conformational states of α-syn along with fully denatured protein. Spectra were acquired using a Synapt G2 HDMS instrument (Manchester, Waters, UK) by use of gold coated home-made borosilicate nano-capillaries in positive mode. Optimized instrumental settings for data acquisition were: capillary voltage of 1.70-1.90 kV, cone voltage of 50 V, source temperature of 60 ^o^C, trap collision energy of 4.0 V, transfer collision energy of 10 V, trap bias 45, backing pressure of 3.1 mbar. IMS separations were performed at T-wave velocities of Trap:311, IMS:800 and Transfer:200 m/s and T-wave amplitudes of 4-15 V using 3.6 mbar pressure of nitrogen gas maintained by a 90 mL/ min gas flow. Native spectra were obtained by dissolving the protein in question to a 40 µM final in aqueous solution of 50 mM ammonium acetate pH 6.8. For attaining denatured spectra, all calibrants and mass standards were prepared before injection at 10-15 µM and α-syn at 10 and 40µM and dissolved in 10% formic acid, 50% acetonitrile and 40% ultrapure water (vol/vol/vol). Calibration curve for Ωs were obtained based on multiple charge states of equine cytochrome c, horse heart myoglobin and bovine ubiquitin (Sigma Aldrich, UK) as described previously [1].

**Table 1. Compiled experimental and theoretical collisional cross sectional areas (Ω) of a range of model proteins.** Experimentally recorded Ω for bovine ubiquitin, equine cytochrome c,
α-lactalbumin, hen egg lysozyme and horse heart myoglobin, native α-synuclein and denatured
α-synuclein reported in Supplementary Figure 1. The reported values are the mean Ω over all charge states of the protein and standard deviations represent the spread in the recorded Ω across all charge states. Theoretical values were calculated by the in-house Leeds algorithm based on their PDB co-ordinates from NMR and X-ray crystallography measurements. The MOBCAL calculations are also shown for the projection approximation (PA) and the exact hard sphere scattering (HS) model [1]. Experimental published values were obtained from references [1] and [2].

**
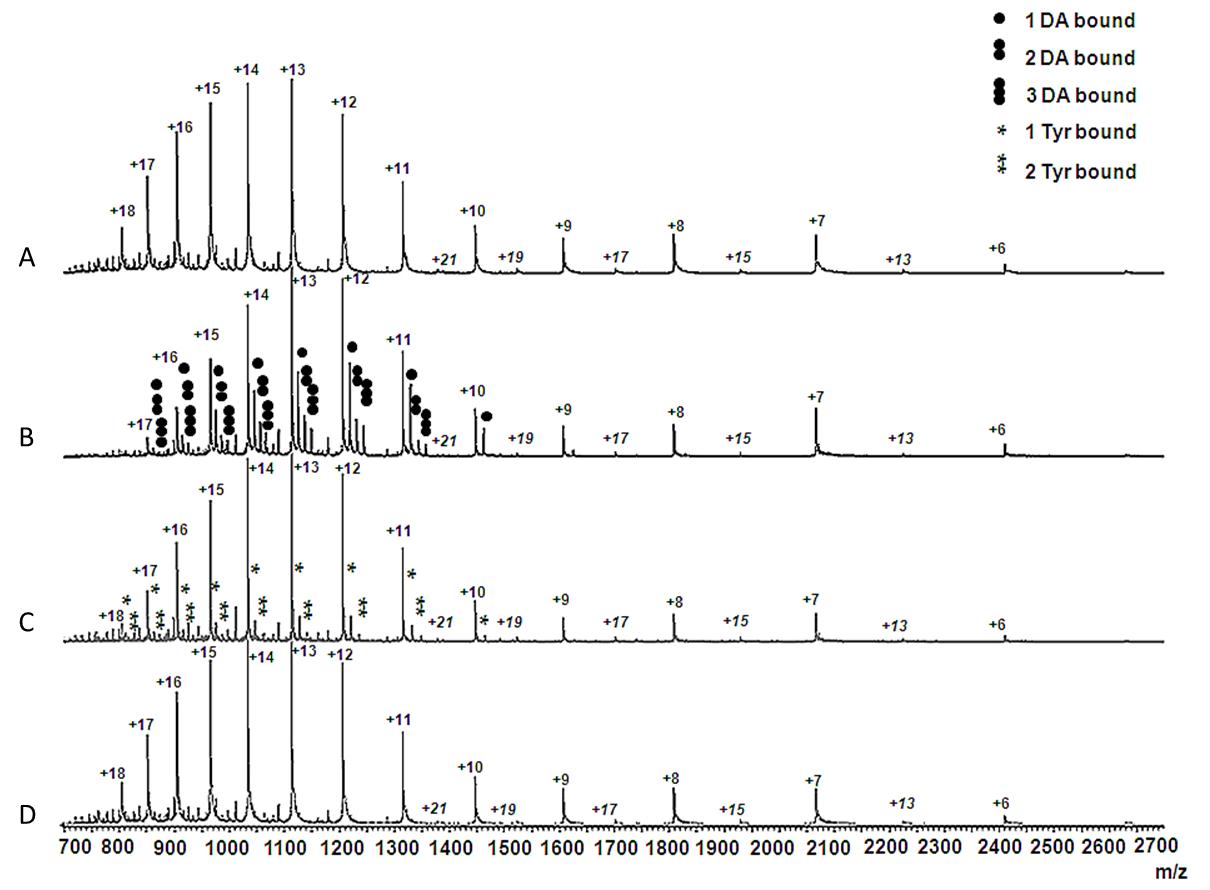
Supplementary Figure 2.**

DA-α-syn complexes observed by ESI-IMS-MS are shown. ESI-MS spectra extracted from the drift scope plots of Figure 1 were all obtained from 50 mM aqueous ammonium acetate solution of 40 µM α-syn, at pH 6.8 with or without further addition of either DA or Tyr at 6.25mM. Charge states and the presence or absence of the appropriate ligands are indicated on the plots. **A.** wild-type α-syn.
**B.** α-syn in the presence of DA.
**C.** α-syn in the presence of Tyr
**D.** α-syn in the presence of DA and Gly.

DA ligands are highlighted with black dots on the mass spectrum in **B**. and are only observed on the +17 to +10. Maximum of two molecules of Tyr are involved in complex formation with α-syn indicated by one or two stars above the respective peaks **C.** these ions however have a markedly lower intensity as compared to apo- and DA bound forms of α-syn.

**
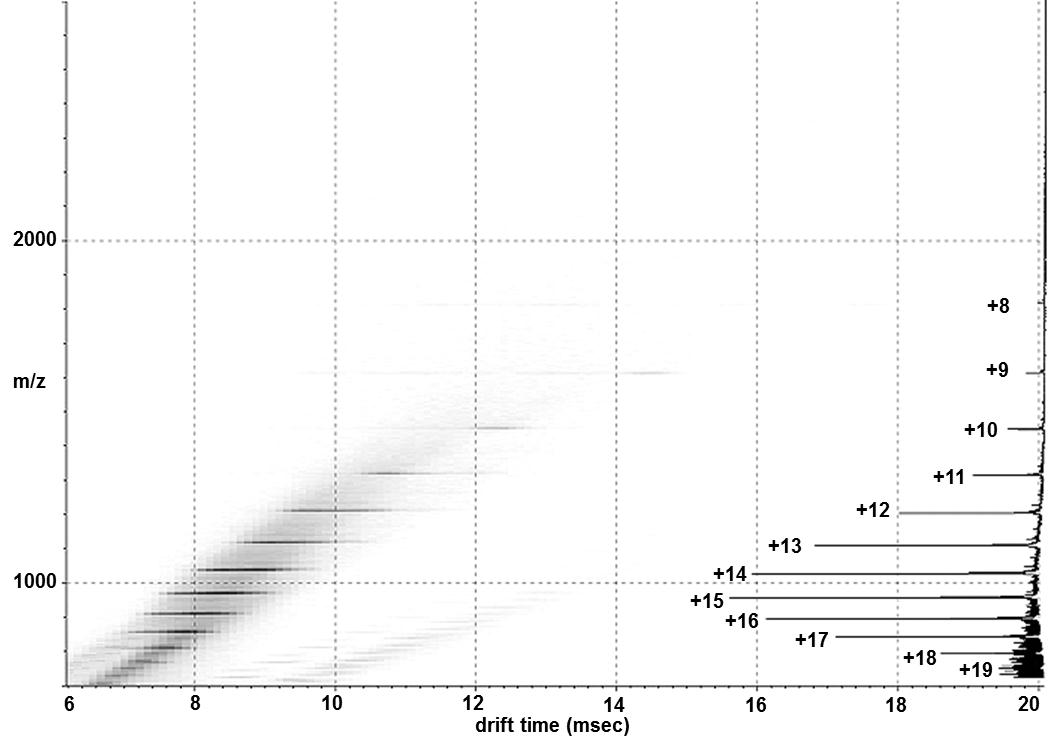
**

**Supplementary Figure 3.** α-syn acquired under denaturing conditions (50% acetonitrile, 40% H_2_O and 10% formic acid) displays only extended conformations. A driftscope plot was acquired using the experimental conditions described above. Charge state ions between +19 and +8 can be observed characteristic of a fully unfolded protein. Charge states associated with the compact state +7 and +6 charge state ions are absent or below the level of detection. We note within the mass spectrum a wide range of very low intensity conformations with little evidence of a defined relatively highly populated compact states at the +9 and +8 charge state ions as observed under native conditions. This spectrum demonstrates that little significant conformational collapse was observed during acquisition and that the spectrum reported here reflects the conformations observed in solution.


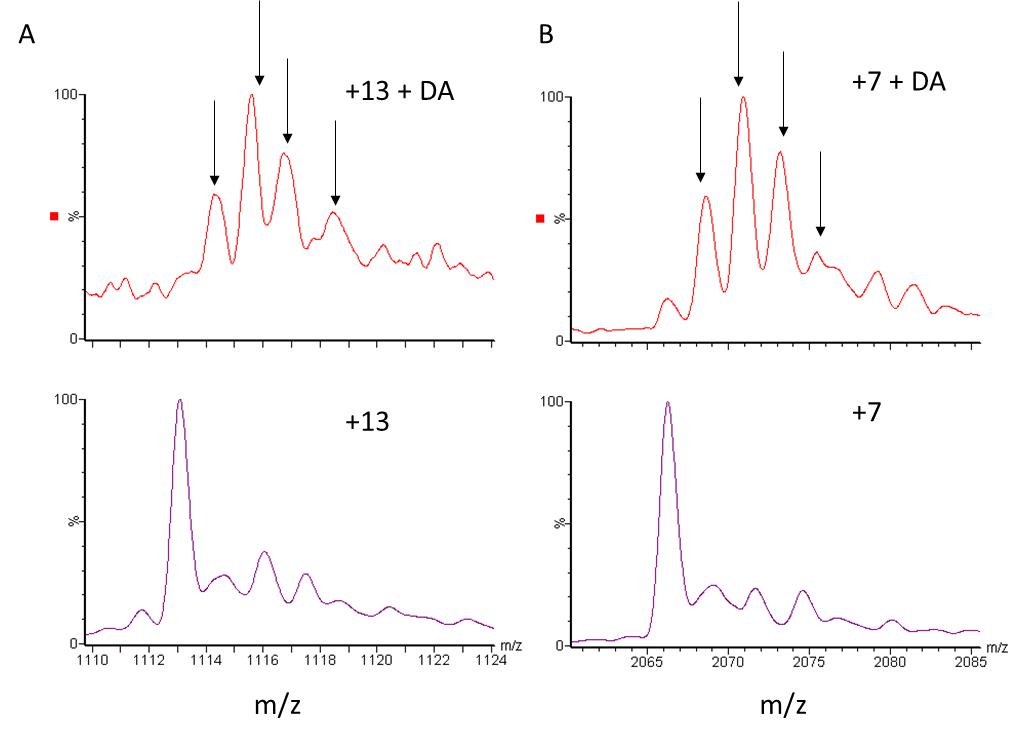


**Supplementary Figure 4.** Oxidation of methionine can be observed on long term exposure of α-syn to DA as reported by others [3]. Samples of α-syn-DA at a 1:8 ratio were allowed to incubate at room temperature without agitation for 12h. The **A.** +13 and **B.** +7 charge state ions are shown, arrows indicate a +16 Da increase in mass consistent with the oxidation of the four methionine residues.

**References**

[1] Smith, D. P., Knapman, T. W., Campuzano, I., Malham, R. W., Berryman, J. T., Radford, S. E., Ashcroft, A. E.: Deciphering drift time measurements from travelling wave ion mobility spectrometry-mass spectrometry studies. *Eur. J. Mass. Spectrom. (Chichester, Eng).* **15**, 113-130 (2009)

[2] Bernstein, S. L., Liu, D., Wyttenbach, T., Bowers, M. T., Lee, J. C., Gray, H. B., Winkler, J. R.: Alpha-synuclein: stable compact and extended monomeric structures and pH dependence of dimer formation. *J. Am. Soc. Mass Spectrom.***15**, 1435-1443 (2004)

[3]Leong, S. L., Pham, C. L., Galatis, D., Fodero-Tavoletti, M. T., Perez, K., Hill, A. F., Masters, C. L., Ali, F. E., Barnham, K. J.: Cappai, R. Formation of dopamine-mediated alpha-synuclein-soluble oligomers requires methionine oxidation. *Free Radic. Biol. Med.* **46**, 1328-1337 (2009)
